# Adaptive STDP-based on-chip spike pattern detection

**DOI:** 10.3389/fnins.2023.1203956

**Published:** 2023-07-13

**Authors:** Ashish Gautam, Takashi Kohno

**Affiliations:** Institute of Industrial Science, The University of Tokyo, Tokyo, Japan

**Keywords:** adaptive STDP, spiking neural networks, 4-bit synapse, mixed-signal neuromorphic chip, spike pattern detection, unsupervised learning, temporal coding, synapse resolution

## Abstract

A spiking neural network (SNN) is a bottom-up tool used to describe information processing in brain microcircuits. It is becoming a crucial neuromorphic computational model. Spike-timing-dependent plasticity (STDP) is an unsupervised brain-like learning rule implemented in many SNNs and neuromorphic chips. However, a significant performance gap exists between ideal model simulation and neuromorphic implementation. The performance of STDP learning in neuromorphic chips deteriorates because the resolution of synaptic efficacy in such chips is generally restricted to 6 bits or less, whereas simulations employ the entire 64-bit floating-point precision available on digital computers. Previously, we introduced a bio-inspired learning rule named adaptive STDP and demonstrated *via* numerical simulation that adaptive STDP (using only 4-bit fixed-point synaptic efficacy) performs similarly to STDP learning (using 64-bit floating-point precision) in a noisy spike pattern detection model. Herein, we present the experimental results demonstrating the performance of adaptive STDP learning. To the best of our knowledge, this is the first study that demonstrates unsupervised noisy spatiotemporal spike pattern detection to perform well and maintain the simulation performance on a mixed-signal CMOS neuromorphic chip with low-resolution synaptic efficacy. The chip was designed in Taiwan Semiconductor Manufacturing Company (TSMC) 250 nm CMOS technology node and comprises a soma circuit and 256 synapse circuits along with their learning circuitry.

## 1. Introduction

The human brain is designated as the most complex thing in the known universe (Herculano-Houzel, 2011). At the microcircuit level, neuronal cells are morphologically arranged in layers with various (mostly unknown) connectivity motifs. However, information processing mechanisms at this level are still not completely understood, and exploring them in known motifs is crucial for developing insights into many aspects, such as the biological mechanisms of learning and the emergence of intelligence.

One of the engineering approaches to understanding the microcircuit of the brain is “analysis by synthesis.” In this bottom-up approach, brain microcircuit models are physically implemented using electronic circuits. Mixed-signal neuromorphic hardware, which has recently gained popularity in “neuromorphic computing,” is another effective tool for understanding the microcircuit (Kohno et al., 2014; Mayr, 2019; Neckar et al., 2019; Pehle et al., 2022). Mixed-signal implementations are more realistic than computer simulations or purely digital implementations. Owing to the thermal noise in silicon, analog neuron circuits inherently generate stochastic spikes (Kohno et al., 2014), similar to neuronal cells, where noise from ion channels and intrinsic neurotransmitter release results in stochastic spiking. Such stochastic spiking is not observed in digital neuron implementations or computer simulations, unless additional noise is incorporated. On the other hand, in purely digital neuromorphic implementation (Davies et al., 2018; Frenkel et al., 2019; Mayr, 2019; Stuijt et al., 2021; Yang et al., 2022), relatively larger scale networks can be implemented as the circuit size can be scaled down with technology node. Additionally, they also have much faster design and testing cycle compared to mixed-signal chips. In this study, we focus on mixed-signal implementation. In addition to energy efficiency and biological plausibility, an extra advantage of this approach is that it can potentially serve as a fundamental technology for utilising information processing in brain microcircuits, either in biomedical applications or in the development of close-to-brain power-efficient artificial intelligence (AI). Regardless of the current limitation in the scalability of mixed-signal implementation, these peculiar advantages make mixed-signal neuromorphic substrates ideal platforms for implementing neuronal networks and exploring biologically plausible learning mechanisms in the near future.

Numerous learning rules have been developed to train SNNs (Diehl and Cook, 2015; Lee et al., 2016; Shrestha et al., 2017; Huh and Sejnowski, 2018; Kheradpisheh et al., 2018; Neftci et al., 2019; Kheradpisheh and Masquelier, 2020; Sakemi et al., 2021). These rules are either inspired by the brain’s mechanisms or are spike-based variants of the backpropagation algorithm, utilising smoothed spike functions or surrogate gradient techniques. This study focuses on the circuit implementation of spike-timing-dependent plasticity (STDP), a commonly observed spike-based learning mechanism in the brain. It has been suggested that a single STDP-empowered neuron can detect spatiotemporal spike patterns embedded in biologically plausible input spike trains (Masquelier et al., 2008). Moreover, it can detect multiple embedded spike patterns using a lateral inhibitory configuration (Masquelier et al., 2009), which is another commonly observed network motif in the brain (Douglas et al., 1989). The input spike trains used in these studies were modelled using an inhomogeneous Poisson process, which is known to capture the basic statistical properties of spiking activity in the brain (Dayan and Abbott, 2001). Spike trains also incorporate noise and jitter into their spike patterns, which roughly correspond to synaptic noise. The embedded spike patterns were solely characterised by their spike timing (rather than spike rate); thus, approximately modelled the temporal coding observed in various neuronal pathways (Thorpe et al., 2001). Since this input model was developed based on biologically possible prerequisites, it has the potential to be a fundamental model for understanding information processing principles in the lateral inhibition network. Another crucial characteristic of the input spike train model used in Masquelier et al. (2008, 2009) is the generality of the embedded spike patterns, making the model agnostic for any particular type of input.

In Masquelier et al. (2008, 2009), synaptic efficacy (weight) was a 64-bit floating-point value. The performance of the spike pattern detection depends on its resolution (high resolution provides better performance). However, the resolution of these non-volatile efficacy variables in a physical implementation is generally limited. In neuromorphic chips developed for neuromorphic computing tasks (for example, MNIST classification) or the neuroscience focused “analysis by synthesis” framework, synaptic efficacy is generally stored using one of three methods: utilising capacitors (Azghadi et al., 2014a), employing digital memory (Schemmel et al., 2006; Moradi and Indiveri, 2014; Thakur et al., 2018) or employing non-volatile memory devices (Kuzum et al., 2013). Analog circuits with capacitor-based efficacy storage are extremely energy efficient, but they suffer from leakage issues, resulting in gradual memory loss over time. Another approach is to use palimpsest synapse circuits that have two stable states in the long term (Indiveri et al., 2006). They overcome the leakage problem, but have a low efficacy resolution (~1.5 bits). Mixed-signal STDP circuits store multibit synaptic efficacy in digital memory and use a digital-to-analog converter (DAC) to convert the efficacy into a synaptic current. However, synapse circuits with high-efficacy resolution require large DAC circuits, limiting the number of synapses that can be implemented on a chip. Since the area of a single-synapse circuit doubles for every one-bit increase in resolution, it is impractical to implement high-resolution synapses. Most chips implement synapses with a resolution between four to six bits. The final approach involves the use of novel non-volatile memory devices (Saxena et al., 2017; Mulaosmanovic et al., 2020). These are still being researched and are believed to be potential solutions for implementing high-resolution synaptic efficacy in a small area. However, a reliable efficacy greater than three bits has not yet been observed in these devices, and they incur hardware implementation overheads upon maturation. For example, ferroelectric field effect transistor (FeFET)-based synapses require relatively high-voltage (>2.5 V) pulses to program their efficacy.

The resolution of individual synapses in the brain remains a topic of debate (Petersen et al., 1998; Enoki et al., 2009; Liu et al., 2017). However, similar to neuromorphic chips, physical synapses in the brain may also face the problem of implementing a high-resolution synaptic efficacy.

It has been established that synaptic efficacy modifications are affected not only by STDP and other Hebbian-based learning rules but also by other factors, such as network oscillations (Hölscher et al., 1997; Hyman et al., 2003) and the presence of neuromodulators (Frémaux and Gerstner, 2015; Andersen et al., 2017). For example, dopamine, a neurotransmitter, has been demonstrated to vary the STDP learning window towards potentiation, regardless of the spike order (Zhang et al., 2009). Inspired by this observation, a hardware-friendly and biologically possible variation of the STDP rule, called adaptive STDP, was proposed in Gautam and Kohno (2021). Using numerical simulations of ideal models, it was shown that the adaptive STDP rule with 4-bit synapses achieves a performance similar to that of the ideal model (64-bit floating-point) for spike pattern detection by a single neuron Gautam and Kohno (2021). In the adaptive STDP rule, the parameter controlling the time window for long-term depression (LTD) is increased during learning. This stabilises the learning process by controlling the learning rate. The efficacy update is also restricted to a single bit at any instant in time by using a rectangular STDP learning window instead of an exponential one, which considerably simplifies circuit implementation.

In this study, we present a circuit to implement the adaptive STDP rule and solve the same problem on a mixed-signal neuromorphic chip. Our results demonstrate that the on-chip performance of the adaptive STDP rule in the presence of fabrication mismatch and thermal noise is similar to that of the numerical simulation of the ideal circuit model. In other words, the performance of the circuit matches that of the ideal model. To the best of our knowledge, this is the first study that demonstrates a mixed-signal neuromorphic chip that can perform spatiotemporal pattern detection, where spike patterns are characterised by spike timings, instead of spike rates, and learning is purely unsupervised using STDP-based rules. The chip was designed in the Taiwan Semiconductor Manufacturing Company (TSMC) 250-nm CMOS process and comprises only 256 synapse circuits (with 4-bit efficacy resolution) activating a biomimetic soma circuit. This relatively large process node was selected owing to its availability and budget constraints. The chip has a single neuron circuit, and we restricted this study to a single neuron-single pattern case. Similar to the STDP rule, the adaptive STDP rule is easily scaled to multiple neurons-multiple patterns case using lateral inhibitory connections between multiple neurons. Its simulation results are found in Gautam and Kohno (2023).

The remainder of this paper is organised as follows. Next section explains the models and experimental setups, followed by a description of the overall architecture and major components of the chip. The biomimetic neuron circuit is not described in this study, and its details are available in Kohno and Aihara (2016) and Kohno et al. (2017). In the Results section, the experimental results of the on-chip spike pattern detection using a single neuron are presented. The final section presents a discussion of the results and conclusions derived from the study.

## 2. Materials and methods

### 2.1. Models and setups

The model is based on a previous study (Masquelier et al., 2008), in which a noisy spatiotemporal spike pattern repeatedly present at irregular intervals in stochastic spike trains was detected by a neuron using STDP learning. The neuron receives spike trains *via* $N_{\mathrm{aff}}$ synapses, where $N_{\mathrm{aff}}$ represents the number of afferents. These spike trains were generated independently *via* an inhomogeneous Poisson process. The instantaneous firing rate was varied between 0 and 90 Hz, and a minimum time of 50 ms was chosen for the spike rate to change from 0 to 90 Hz. Each afferent spike occurred at least once within a 50 ms duration, fixing 20 Hz as the minimum spiking frequency. Once the stochastic spike trains (225 s long) for $N_{\mathrm{aff}}$ synapses were generated, a 50 ms long slice (the spike pattern to be detected) was randomly chosen and copied. The original spike train was then divided into 50-ms-long sections and constrained by the desired spike pattern appearance rate (chosen to be 25 or 10%); a certain number of these sections were randomly chosen and replaced by the spike pattern to be detected. During the copy-and-paste process, consecutive 50 ms sections were avoided. The population-averaged firing rate of these afferents in 10-ms time -bins was approximately the same throughout the input spike train (approximately 54 Hz). The 50-ms sections comprising the spike patterns also have the same population average spike rate as the rest of the input spike train. The presence of spike patterns is characterised by nothing other than the specific spike times of the afferents. Subsequently, an additional 10 Hz spontaneous noise was added to the spike trains of all the afferents to increase the difficulty of pattern detection, and a random jitter was introduced in the exact timing of the spike within the spike pattern. In the absence of this additional noise and jitter, all afferents encoding the spike pattern would fire in precisely the same manner in each pattern presentation. The inclusion of the additional 10 Hz noise increased the population average firing rate of the afferents (measured in 10 ms time bins) to approximately 64 Hz. The jitter in the spike timing was modelled using a Gaussian distribution with zero mean and a standard deviation of 1 ms.

In Masquelier et al. (2008), the LIF neuron model was used, and $N_{\mathrm{aff}}$ was 2000, of which only half encoded the spike patterns. The resolution of synaptic efficacy was employed using 64-bit floating point available on digital computers, and the ideal STDP rule biassed towards depression was used. The chip used to demonstrate the results in this study has a qualitatively modelled biomimetic neuron circuit, and $N_{\mathrm{aff}}$ was reduced to 256 because its integrated circuit technology node (250 nm) was too large to integrate 2048 synapse circuits in the available chip area. The adaptive STDP rule was used, the resolution of the synapses was restricted to four bits, and the update in synaptic efficacy at any instant was restricted to a single bit.

**Figure 1:**
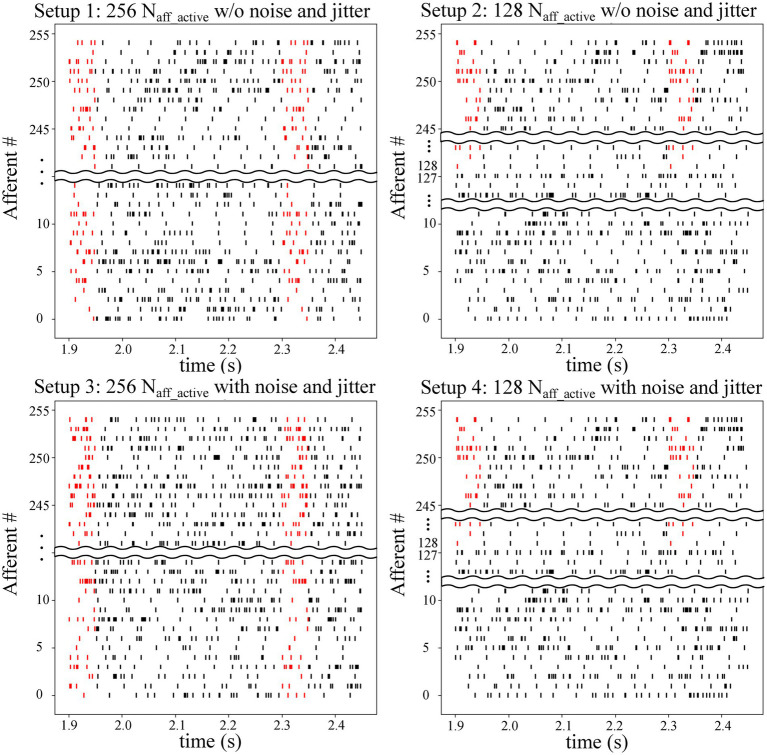
Raster plot of afferents in the four setups. Embedded spike patterns are highlighted in red. Setups 1 and 3 use 256 afferents to encode the spike patterns, whereas Setups 2 and 4 only use half of the afferents (128 out of 256). Afferents in Setups 3 and 4 have a jitter (with a standard deviation of 1 ms) in the spike timing within the patterns along with additional stochastic 10 Hz spikes. Average spiking frequency of afferents in these setups is 64 Hz. Setups 1 and 2 do not have this additional noise and jitter and have an average spiking frequency of 54 Hz. Spike patterns are temporally coded. More specifically, the spike patterns are only characterised by the spike timing of the afferents. Spiking rate inside and outside the pattern is the same.

On-chip experiments were conducted using four different setups. A summary of the experimental setups is provided in Table 1, and raster plots of the input spike trains for all four setups are shown in Figure 1. In Setups 1 and 3, all 256 synapse circuits were activated using stochastic spike trains comprising hidden spike patterns. The only difference is that the input spike trains in Setup 3 have additional 10 Hz Poisson spikes and random jitters in spike timing within each instance of the spike pattern. The jitters were modelled as a Gaussian random variable with zero mean and a standard deviation of 1 ms. In Setups 2 and 4, only 128 of the 256 afferents were used to encode the repeating spike patterns, whereas the remaining 128 encoded only Poisson spikes. In other words, only half of the total afferents encode the spike patterns. In Table 1 and Figure 1, $N_{\mathrm{aff}\_\mathrm{active}}$ represents the number of afferents actively encoding the pattern. In addition, similar to Setup 3, the spike trains in Setup 4 comprised the aforementioned additional noise and jitter. Setups 2 and 4 demonstrated applicability in more practical cases, where the repeating spike pattern may not be encoded by all afferents. Compared with the reference study (Masquelier et al., 2008), the number of afferents was significantly reduced (from 2048 to 256), which made pattern detection more challenging. Hence, additional noise and jitter were not included in Setups 1 and 2 to compensate for this change. The input spike trains in all setups were 225 s long, and 50 runs were executed for each setup. In Masquelier et al. (2008), a 225 s input spike train was repeated twice to make it 450 s long. However, since most of the learning takes place in the initial phase of the run, we used a 225 s long input in this study.

**Table 1:** Summary of the experimental setups for on-chip learning with adaptive STDP rule.

Setup	1	2	3	4
Number of afferents ($N_{\mathrm{aff}}$)	256	256	256	256
Number of active afferents ($N_{\mathrm{aff}\_\mathrm{active}}$)	256	128	256	128
Additional noise and jitter	NO	NO	YES	YES

### 2.2. Circuit description

#### 2.2.1. Overall architecture

**Figure 2:**
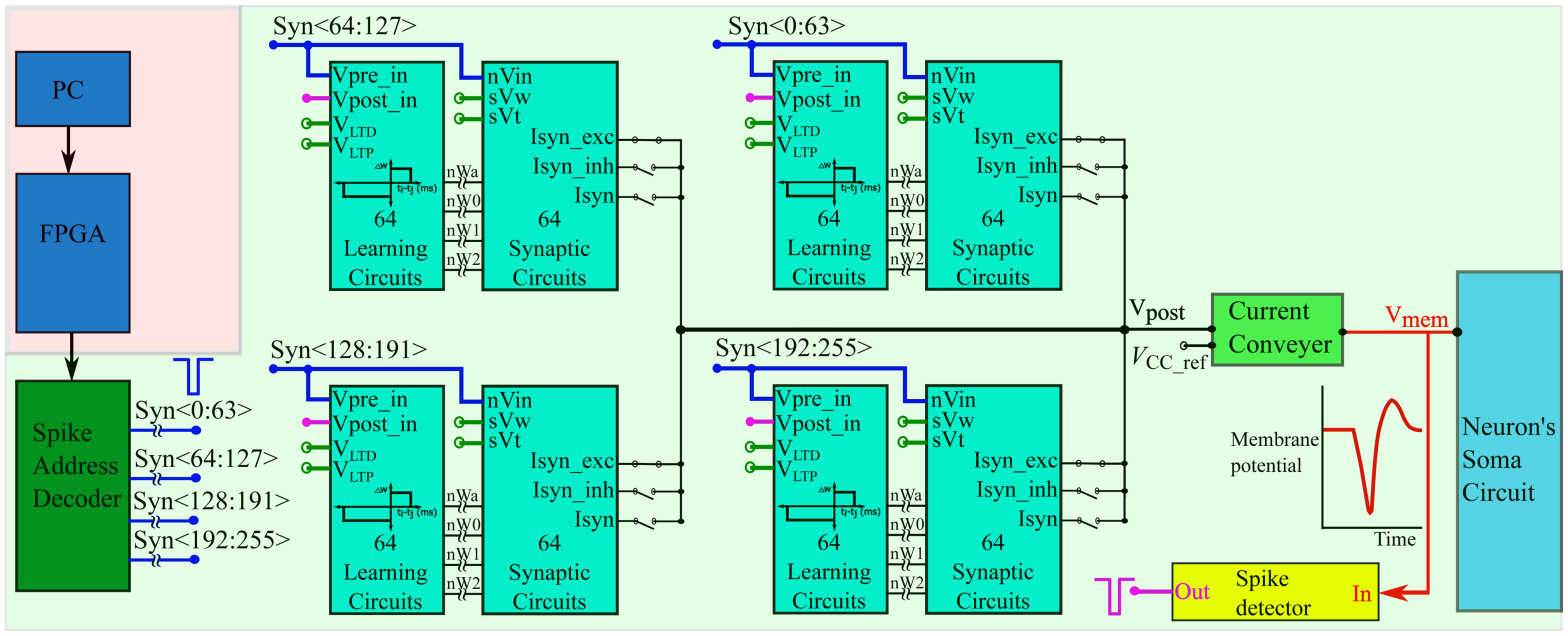
Block diagram of the spike pattern detector. The spike trains are transmitted from the PC to the spike address decoder circuit of the chip *via* an FPGA. The chip comprises one neuron circuit with 256 synapse circuits. Each synapse has 4-bit efficacy resolution and a learning circuitry. The voltages *V*_LTD_, *V*_LTP_, *sV*_w_, and *sV*_t_ are applied *via* external voltage sources and are common to all 256 synapse/learning circuits. A current conveyor circuit is used as an interface between the soma and synapse circuits and the bias voltage Vcc_ref (also applied *via* an external voltage source) sets the node voltage Vpost to approximately the same value *via* the feedback action of the current conveyor circuit. All voltage nodes with open circles are connected to external voltage sources.

**Figure 3:**
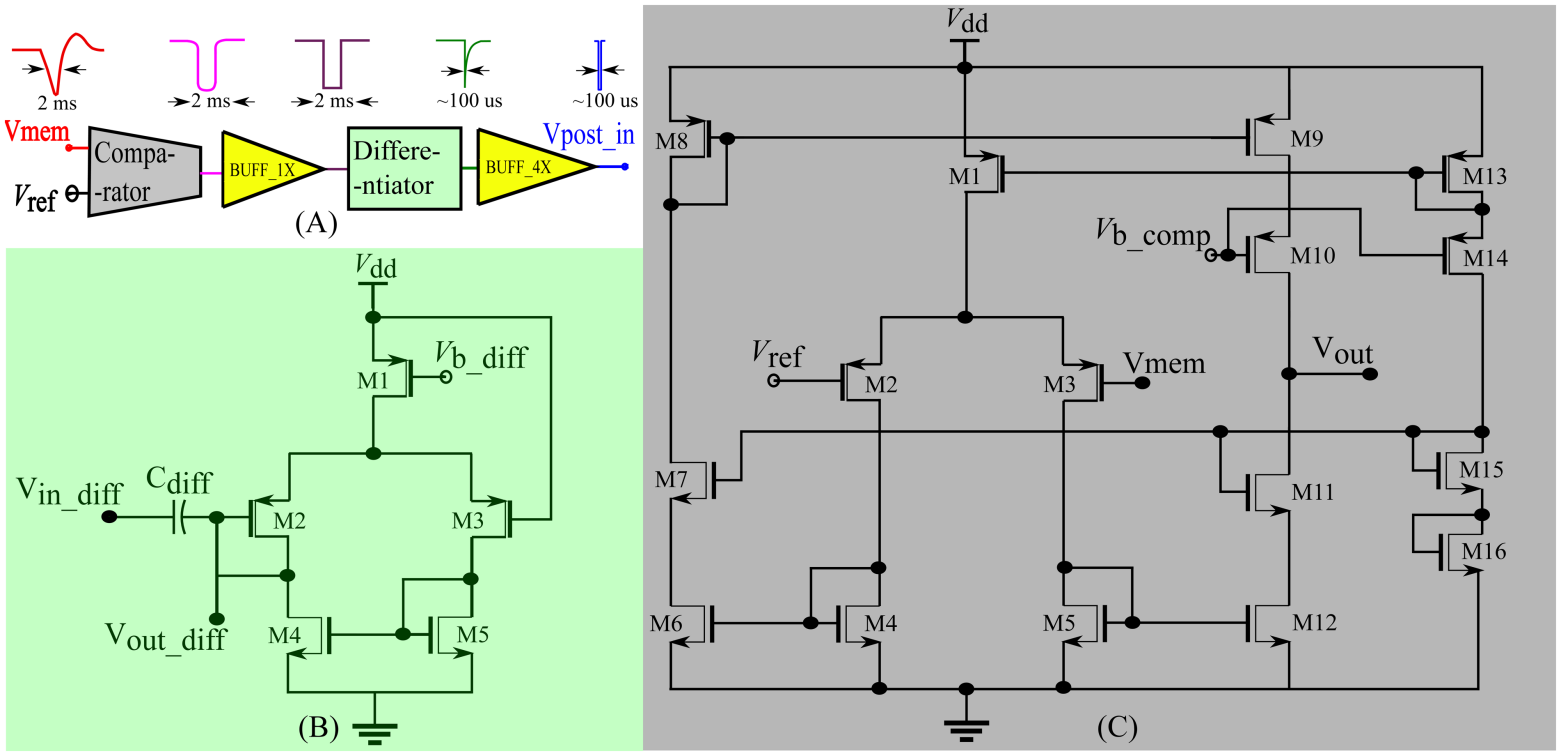
Spike detector circuit shown in Figure 2. **(A)** Block diagram of the circuits with sample voltage output waveforms for each block. BUFF_1x comprises two inverters connected in series and BUFF_4x comprises four inverters with successively increasing width connected in series to drive the node Vpost_in. **(B)** Differentiator circuit **(C)** Comparator circuit.

The input spike trains are transmitted from the PC to the chip *via* a field-programmable gate array (FPGA). An on-chip spike-address decoder circuit receives the target address of the synapse and activates it asynchronously. A block diagram of the chip’s circuits used for spike pattern detection is shown in Figure 2 (green-shaded region). It has a single neuron comprising a biomimetic soma circuit that receives currents from 256 excitatory synapses *via* a bidirectional current conveyor circuit (Chaisricharoen et al., 2010; Kohno and Aihara, 2016; Kohno et al., 2016). The neuron circuit implements a point neuron model, and a current conveyor circuit is required as an interface, because if the synapse circuits are directly connected to the soma circuit, their high parasitic capacitance and leakage current distort the spiking dynamics of the soma circuit. The current conveyor replicates the currents induced by the synapses into the soma, and thus implements the single-compartment point neuron model. The soma was primarily designed using PMOS transistors because they have a significantly lower leakage current than their NMOS counterparts, thereby minimising the overall static power consumption of the circuit. Therefore, its current polarities and spiking behaviour are opposite to the conventional directions, and an excitatory (inhibitory) synapse circuit induces a current out of (into) the soma circuit and depolarises (hyperpolarizes) it. It consumes less than 6 nW of static power and is configurable in several spiking modes, including major neuronal cell classes (e.g., fast-spiking, low-threshold spiking, and regular spiking). In this study, it is configured in the Class 1 mode in Hodgkin’s classification without spike frequency adaptation (fast-spiking), which is qualitatively equivalent to the LIF model. The spikes (action potentials) generated by the soma circuit are approximately 2 ms wide and are converted into pulses by the spike detector circuit (Figure 3A). Its first-stage circuit is a wide-range transconductance circuit configured as a comparator (Figure 3C) that compares the membrane potential of the soma circuit with a fixed voltage ($V_{\mathrm{ref}}$) and outputs a pulse approximately 2 ms wide (similar to the width of the spike). Subsequently, a follower differentiator circuit (Figure 3B) reduces the pulse width to around 100 μs (Mead, 1989). This pulse represents the postsynaptic spike and is fed back to the learning circuitry of all synapse circuits. A multistage buffer is used at the output owing to the high parasitic capacitance of node Vpost_in (connected to the learning circuits of the 256 synapse circuits).

#### 2.2.2. Synapse circuit

**Figure 4:**
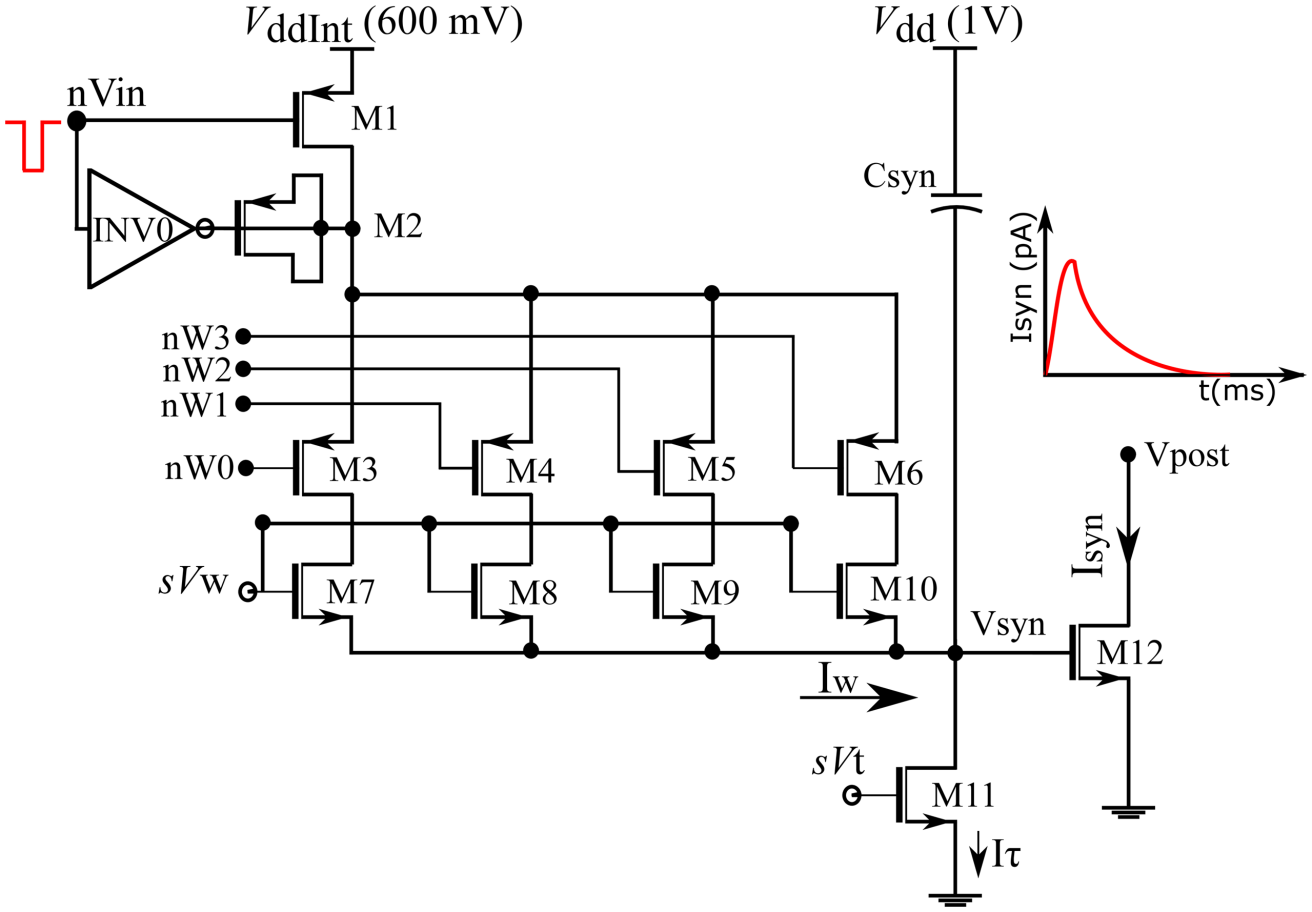
Schematic of excitatory synaptic circuit. Binary-weighted transistors’ dimensions: M7 = 0.3758*(w/l), M8 = w/l, M9 = 2*(w/l) and M10 = 4*(w/l). Efficacy bits nW0-nW3 connect to the learning circuitry.

The fabricated chip comprises 256 synapse circuits with configurable polarity. In this study, all synapse circuits were configured to induce excitatory currents independent of the postsynaptic membrane potential. A schematic of the circuit is shown in Figure 4. It has three stages: a 4-bit DAC (M1–M10) that implements synaptic efficacy, an integrator stage ($C_{\mathrm{syn}}$ and M11) similar to the log-domain integrator (LDI) (Merolla and Boahen, 2004), and an output stage (M12). This circuit is a modified version of the circuit proposed in our previous study (Gautam and Kohno, 2020), in which the output stage comprises a transconductance amplifier circuit instead of a single transistor, M12. Its detailed description is found in Gautam and Kohno (2018, 2020). A brief description of the circuit operation is provided below: Transistors M7–M10 in the DAC stage are binary weighted, and their activation is switched by transistors M3–M6. Their state is controlled by a learning circuit that configures the 4-bit synaptic efficacy (nW0-nW3). The MOS capacitor M2, along with the inverter INV0 form a charge injection compensation module. Bias voltages $sV_{w}$ and $sV_{t}$ control the amplitude scale and time constant of the synaptic current, respectively. The on-chip spike-address decoder circuit transmits a pulse to a synapse upon receiving its address. The input pulse at node nVin activates the synapse’s DAC stage and charges node Vsyn for the duration of the input pulse. Subsequently, Vsyn is linearly discharged by a constant current sunk by M11 operating in the saturation region. The circuit operates in the subthreshold regime and the linear charging and discharging profile of Vsyn induces an exponential current through transistor M12, thus mimicking the standard synaptic current profile. The circuit was designed in the TSMC 250 nm technology node, with each synapse circuit occupying an area of 4,400 μm^2^. The design also includes circuits for other configurations (inhibitory and conductance-based). In this study, synapse circuits were configured to generate excitatory synaptic currents similar to fast AMPA synapses, (Destexhe et al., 1998) with $sV_{w}$ and $sV_{t}$ fixed at 230 and 160 mV, respectively.

#### 2.2.3. Learning circuitry

**Figure 5:**
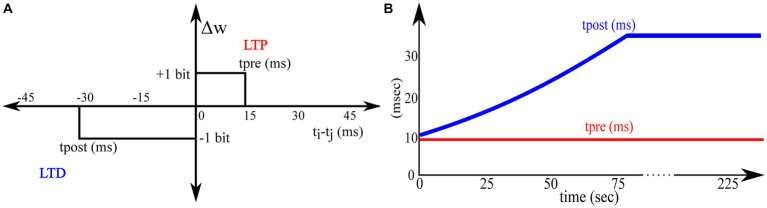
Adaptive STDP learning rule. **(A)** Rectangular STDP function **(B)** Adaptation of tpost while learning.

All the synapse circuits have a learning circuit to implement the adaptive STDP learning rule. Similar to the STDP learning rule, the adaptive STDP learning rule updates the synaptic efficacy based on the spike timings of the pre- and postsynaptic neurons; however, the modification (if any) in the synaptic efficacy is restricted to one bit (the least significant bit). This is described by the learning function shown in Figure 5 and is mathematically expressed as follows:

$$\Delta w_{j}=\left\{\begin{array}{c} +1\;\mathrm{bit},\mathrm{if}\;t_{j}\leq t_{i}\;\mathrm{and}\;t_{i}-t_{j}<t_{\mathrm{pre}}\;\left(LTP\right),\\ -1\;\mathrm{bit},\mathrm{if}\;t_{j}>t_{i}\;\mathrm{and}\;t_{j}-t_{i}<t_{\mathrm{post}}\;\;\left(LTD\right), \end{array}\;\left(1\right)\right.$$

**Figure 6:**
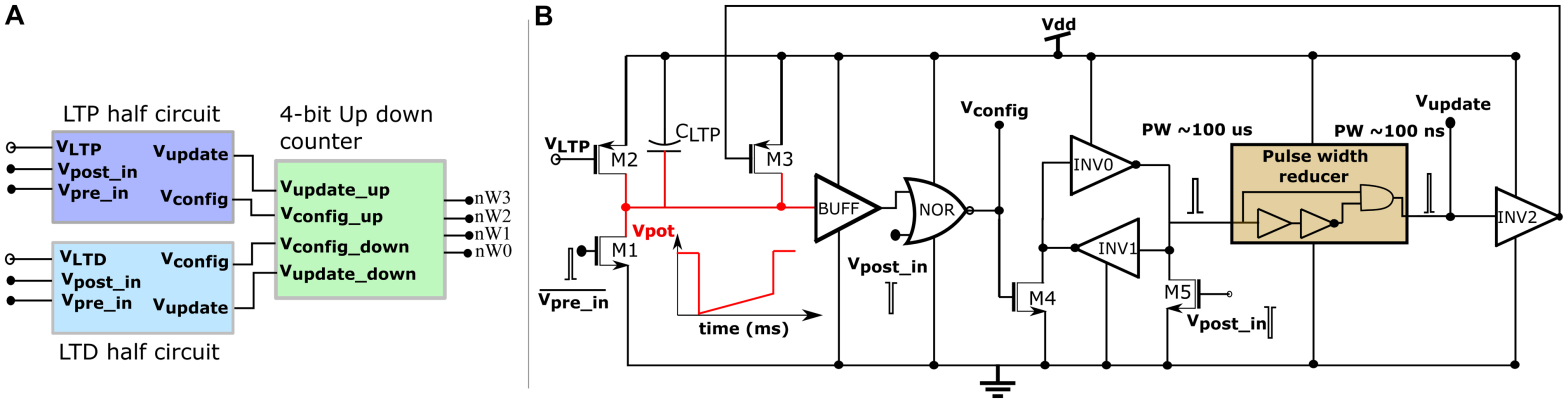
Learning circuitry. **(A)** Top level block diagram **(B)** Schematic of the LTP half-circuit controlling potentiation.

Where $t_{pre}$ denotes the maximum delay of the postsynaptic spike after the presynaptic spike that leads to potentiation (LTP), $t_{post}$ denotes the maximum delay of the presynaptic spike after the postsynaptic spike that leads to depression (LTD), and all the other variables have their general meanings. The learning parameter $t_{pre}$ is kept constant during learning, and $t_{post}$ is increased, as shown in Figure 5B. A detailed description of this learning rule is presented in Gautam and Kohno (2021). A block diagram of the learning circuit used for implementing adaptive STDP learning is illustrated in Figure 6A. The circuits in the block LTP and LTD are symmetric, and calculate the time difference between pre-and postsynaptic spikes according to (1) and potentiate or depress the synaptic efficacy. The synaptic efficacy is stored in a 4-bit up-down counter that saturates at its maximum (15) and minimum (0) values. A conceptual schematic of the half-circuit controlling potentiation (LTP) of the synaptic efficacy is shown in Figure 6B. The potentiation and depression half-circuits are symmetric. In the latter half-circuit, the terminals $\bar{Vpre\_in}$ and Vpost_in are interchanged. To update the synaptic efficacy, the counter receives two successive pulses from each half-circuit: A configuration pulse (Vconfig) and an update pulse (Vupdate). When arriving from the LTP (LTD) half-circuit, the former pulse configures the counter to count up (down), and the latter pulse potentiates (depresses) the counter value. The output of the 4-bit counter is connected to the DAC stage of the synapse circuit, i.e., to terminals nW0- nW3 shown in Figure 4.

The potentiation half-circuit (Figure 6B) operates as follows: A presynaptic pulse at $\bar{\mathrm{Vpre}\_\mathrm{in}}$ activates M1 and discharges node Vpot which then pulls down the top terminal of the NOR gate (*via* the buffer BUFF). When a postsynaptic pulse arrives soon (within $t_{\mathrm{pre}}$ ms at Vpost_in) after the presynaptic pulse, both terminals of the NOR gate are pulled low and node Vconfig goes high for the duration of the postsynaptic pulse (~ 100 μs wide). Consequently, transistor M4 switches on and swaps the state of the latch (INV0 and INV1) whilst generating a pulse (~ 100 μs wide) at the input node of the pulse width reducer circuit. This reduces the input pulse width and generates a pulse (~ 100 ns) at the output node Vupdate. Signals Vconfig and Vupdate modify counter value. First, Vconfig configures the state of the up-down counter to count up, and then, Vupdate increases its count value. However, when the delay between the pre- and postsynaptic pulses is greater than $t_{\mathrm{pre}}$, transistor M2 charges node Vpot back to $V_{\mathrm{dd}}$. Therefore, the output of the NOR gate does not flip to a high state even when a postsynaptic pulse arrives and the counter value remains unchanged. The drain current of M2 is set by the bias voltage $V_{\mathrm{LTP}}$ which, along with the value of the capacitor $C_{\mathrm{LTP}}$ decides$\;t_{\mathrm{pre}}$ in the adaptive STDP rule (1). The higher the value of$\;V_{\mathrm{LTP}}$, the higher the value of$\;t_{\mathrm{pre}}$. The inverter INV5 at the output node (Vupdate) resets node Vpot once the counter is potentiated. This ensures that only the most recent pair of pre- and postsynaptic spikes is considered to update the synaptic efficacy [as per the learning rule implemented in Masquelier et al. (2008)], instead of considering the entire history of spikes. In the experimental setups, $V_{\mathrm{LTP}}$ was fixed at 780 mV. The initial value of $V_{\mathrm{LTD}}$was fixed close to that of $V_{\mathrm{LTP}}$ at 783 mV and later adapted to higher values during learning, as shown in Figure 5B. A higher $V_{\mathrm{LTD}}$ implies a higher value of$\;t_{\mathrm{post}}$. The current chip did not contain adaptation circuitry, and the adaptation of $V_{\mathrm{LTD}}$ was controlled using an external voltage source.

#### 2.2.4. Bidirectional current conveyor circuit

**Figure 7:**
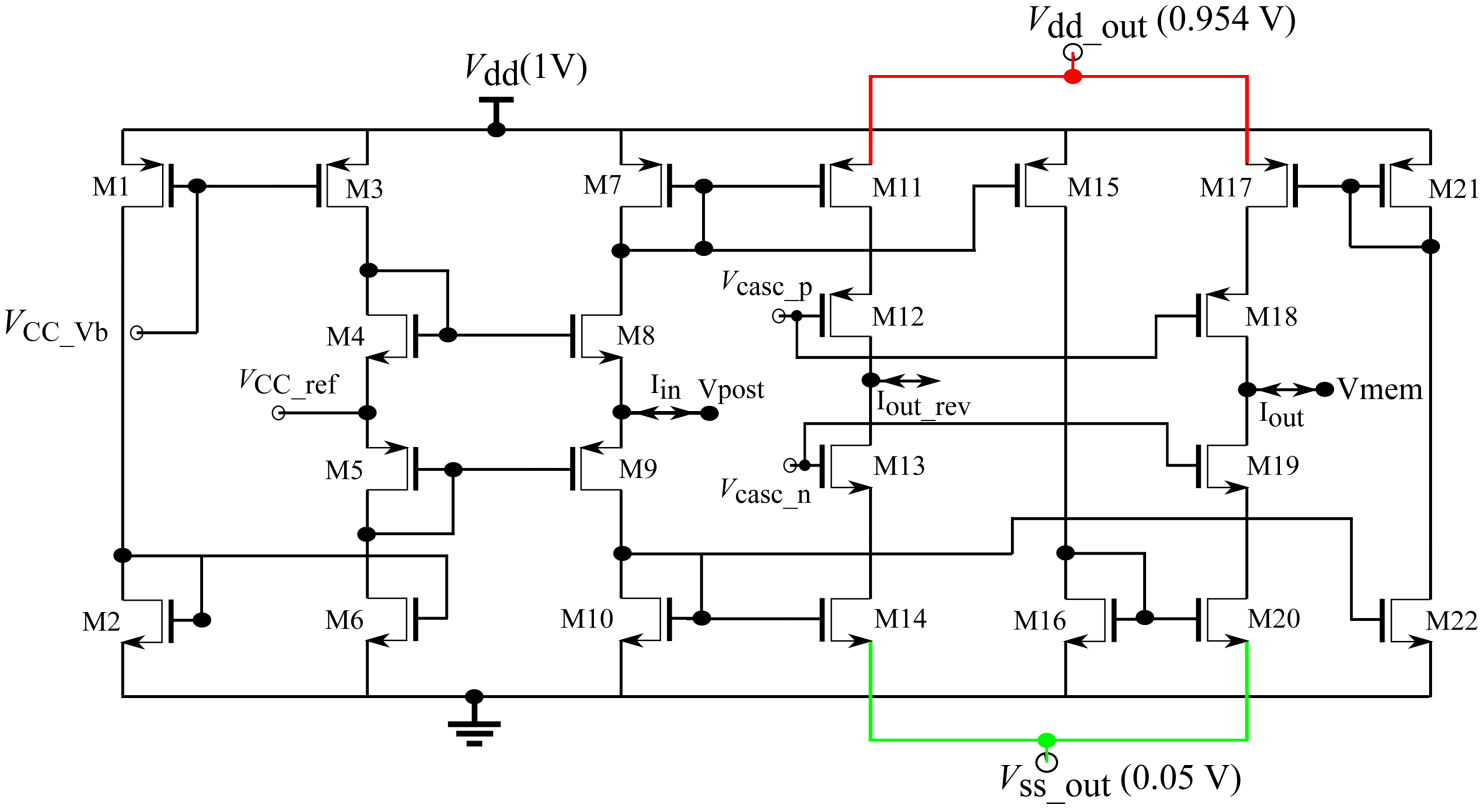
Current conveyor circuit.

A standard bidirectional current conveyor circuit (Figure 7) was used to connect the soma and synapse circuits (Chaisricharoen et al., 2010). Its input node, Vpost, connects to the output of 256 synapse circuits, and its output node, Vmem, connects to the soma circuit. Upon the activation of the synapses, a current is drawn out of node Vpost. This current is sourced by M7, mirrored into M15, sunk by M16, mirrored into M20, and drawn out of node Vmem *via* cascoded transistor M19. The current conveyor circuit conveys the current drawn from its input node to its output node, Vmem, and depolarises the soma circuit. Voltage $V_{\mathrm{CC}\_\mathrm{ref}}$ (600 mV) fixes the voltage of node Vpost to approximately the same value as its own *via* the feedback generated by transistors M4, M5, M8, and M9. The voltage bias $V_{\mathrm{CC}\_\mathrm{Vb}}$ was fixed at 630 mV. The power supply rails of the output branch $V_{\mathrm{dd}\_\mathrm{out}}$ and $V_{\mathrm{ss}\_\mathrm{out}}$ were set at slightly lower and higher voltages than their ideal values of 1 and 0 V, respectively, to minimise the thermal noise induced by the circuit into the neuronal soma (See Discussion).

#### 2.2.5. Spike train transfer

Stochastic spike trains with hidden spike patterns to be detected (generated using the procedure described in Section 2.1) were used to activate the synapse circuits. They were transmitted from a PC to a chip in real time *via* a FPGA. The transmitted spike train data comprises the addresses of the target synapse circuits (0 to 255) along with their activation times. An on-chip spike-address decoder asynchronously activates the synapse circuits upon receiving their address. The FPGA was used to implement first-in-first-out (FIFO) logic that stores the addresses of the synapse circuits and their activation times (received from the PC). The activation times are transmitted and stored as the relative time differences between subsequent input spikes in the incoming spike train. The spike address decoder circuit receives the address of the synapse circuit from the FIFO logic in the FPGA and instantly activates it. A single-address bus connects the FIFO output of the FPGA to the spike address decoder. FPGA measures time (in steps of 10 μs). When the measured time matches the activation time in the FIFO output, it loads the corresponding address onto the address bus connecting the FIFO and the spike address decoder. The decoder is a high-speed circuit that activates the synapse circuit in less than 20 ns upon receiving its address and generates a 2 ms wide pulse to activate the desired synapse circuit. When the activation times of two or more synapse circuits overlap, they are activated sequentially with a 10 μs delay. In a typical run, a maximum error of 50 μs was observed owing to such overlaps. On average, in the input spike train, the time difference between the activation times of any two synapse circuits is in the order of 80 μs, and the error of 50 μs (overlap in the activation time of five synapse circuits) is an extreme case that occurs rarely. The timescale of the soma and synapse circuits is in the order of milliseconds. Hence, activation time errors in the order of 10 of microseconds can be ignored.

## 3. Results

The on-chip experiments (for the setups listed in Table 1) were performed in two groups. The input spike trains in the first (second) group contain a 50 ms long spike pattern to be detected with a 25% (10%) appearance rate. In other words, in the first group, approximately 1,125 spike patterns were hidden in 225 s long spike trains, and in the second group, approximately 450 spike patterns were hidden. The second group had more stochastic spikes (90 vs. 75%) than the first group, which made the pattern detection more challenging. The on-chip performance of the adaptive STDP learning rule is summarised in Table 2. The success criteria was chosen to be a hit rate (neuron spikes within the pattern) greater than 98% and zero false alarms (neuron spikes outside the pattern) in the last 75 s (one-third duration) of the run, which is similar to the criteria used in studies (Masquelier et al., 2008; Gautam and Kohno, 2021).

**Table 2:** Experimental results of adaptive STDP rule.

Setup	1	2	3	4
Success rate with 25% pattern appearance rate.	96%	80%	88%	26%*
Success rate with 10% pattern appearance rate.	90%	64%*	80%	14%*

### 3.1. Setups 1 and 3

**Figure 8:**
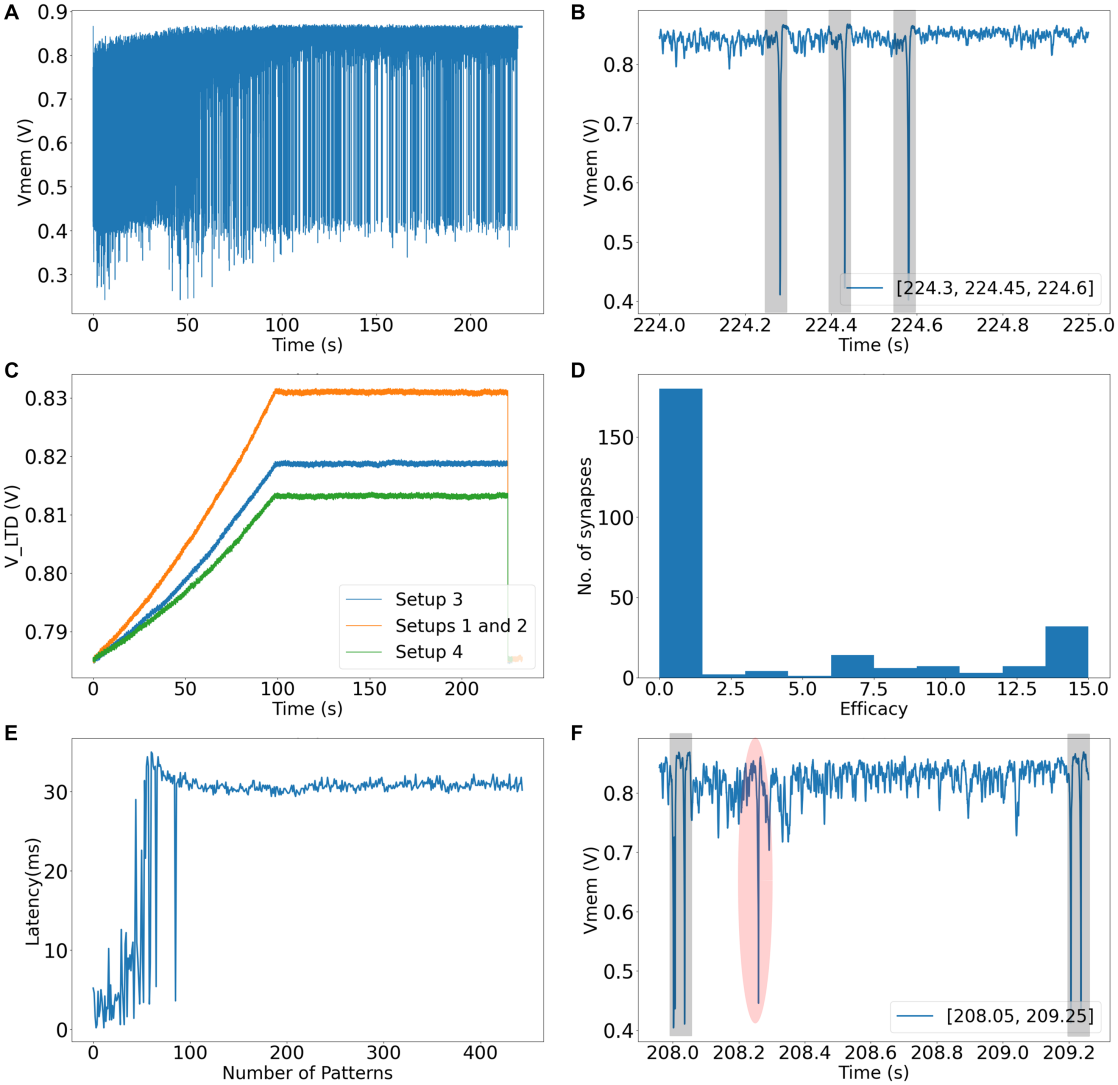
**(A)** Soma circuit’s membrane potential during the run. **(B)** Soma circuit’s membrane potential during the last second, it spikes within the shaded 50 ms spike pattern. **(C)** The adaptation in the value of *V*_LTD_ while learning (shown for all setups). **(D)** Bimodal distribution of synaptic efficacies after learning. **(E)** Latency to spike within the 50 ms pattern. **(F)** An instance of failed run showing false alarm (shaded red) and double spikes within the spike pattern (shaded grey).

In Setup 1 (3), success rates of 96 (88) and 90 (80) % were obtained for spike pattern appearance rates of 25 and 10%, respectively. Both setups used 256 afferents to encode the spike patterns. The performance of Setup 3 is worse than that of Setup 1, owing to the presence of additional noise and jitter. In the majority of runs that did not meet the success criteria in Setup 3, learning occurred. However, there were false alarms and (or) the hit rates were less than 98%. The false alarms had low amplitudes and were visibly different from the regular spikes within the pattern (Figure 8F). Amongst the runs that had the highest number of false alarms (> 10), the neurons spiked twice within the 50 ms pattern, thereby implying that too many synapses were potentiated which likely caused numerous false alarms. A detailed breakdown of the performance of all the setups is presented in Table 3. The failed run column represents cases where the neuron stopped spiking during the learning process. An example of time evolution of the neuron dynamics in a successful run in Setup 3 with a pattern appearance rate of 10% is shown in Figures 8A–E. The spiking behaviour of the soma is shown in Figure 8A. A high spiking frequency was initially observed, which decreased as learning progressed, and the neuron became more selective to spike inputs. The spiking behaviour of the neuron in the last second is shown in Figure 8B. As expected, the neuron spiked only in the presence of this pattern. The times at which the 50 ms pattern ends are superimposed in the bottom-right corner of the figure, and the pattern durations are marked by grey boxes. Figure 8C shows the adaptation of the $V_{\mathrm{LTD}}$ during training. Three adaptation curves corresponding to Setups 1 and 2 (orange curve), Setup 3 (blue curve), and Setup 4 (green curve) are plotted. Figure 8D shows the bimodal distribution of the synaptic efficacy after the completion of the run. Figure 8E shows how the time required to spike within a pattern changes during learning. In this run, the neuron learned to spike within approximately 30 ms. The final figure shows an instance of double spikes within the pattern, and a false alarm from an unsuccessful run. The false alarm profile was markedly different (low in amplitude with non-existent refractory action) from the spikes that occurred within the pattern.

**Table 3:** Performance breakdown showing number of runs for each setup.

Setups with pattern appearance rate		100% hit rate and 0 false alarms	100% > hit rate > 98% and 0 false alarms	>98% hit rate and 1 < false alarms<40	98% > hit rate > 94%	Failed runs	Total runs
1	25%	48	–	1 (1 false alarm)	–	1	50
	10%	44	–	6 (< 5 false alarms)	–	0	50
2	25%	40	–	5 (<3 false alarms)	–	5	50
	10%	32		6(<3 false alarms)		12	50
3	25%	35	9	1 (<5 false alarms)	4 (0 false alarms)	1	50
	10%	31	9	4 (< 30 false alarms, and double spikes)	2 (0 false alarms)	4	50
4	25%	2	11	8 (<5 false alarms) 6 (< 40 false alarms)	6(<10 false alarms)	17	50
	10%	3	4	10 (<5 false alarms) 3 (<40 false alarms)	3(<4 false alarms)	27	50

### 3.2. Setups 2 and 4

In Setups 2 and 4, only half of the afferents encoded the spike patterns (with additional noise and jitter in setup 4). These results are not deterministic. Different results were observed for the same input over multiple trials: some runs were successful, whereas others failed. Specifically, when the pattern appearance rate was 10% in Setup 2 and it was 10 and 25% in Setup 4. Two types of variations were observed: the hit rate varied, and the neuron stopped spiking whilst learning. The success rates (marked with *) listed in Table 2 are based on the first run for each of the 50 input-spike trains. In Setup 2 (10% pattern appearance rate), the neuron either stopped spiking during learning or successfully learned to detect the pattern with a 100% hit rate in multiple trials with the same input. In Setup 4 (25% pattern appearance rate), significant variations were observed primarily in the hit rate. In some trials (with the same input), hit rates greater than 98% were achieved, whereas their values were much lower (in the range of 80 to 98%) in the others. Both types of variations were observed in Setup 4 (10% pattern appearance rate). We attribute this behaviour to the thermal noise in the chip, as fixed-pattern noise or second-order effects by themselves cannot give rise to this probabilistic behaviour. Since Setups 1 and 3 employed a higher number of afferents, their performances were immune to the effect of thermal noise. The worst performance was obtained in Setup 4 because its spike pattern model was the most difficult (128 active afferents with additional noise and jitter in the spike patterns).

### 3.3. Ideal model vs. on-chip performance

To ensure a fair comparison, ideal model simulations were performed using the same input spike trains across all setups. The performance comparison is tabulated in Table 4. The performance of on-chip pattern detection was better than that achieved in the ideal model simulation, which is surprising since the latter is expected to have better performance intuitively. However, most of the failures in the ideal model simulation were due to presence of a few false alarms. If the success criterion is slightly relaxed to allow false alarms (less than 1%), the performance of ideal model simulation comes close to that of the on-chip experiments. The complexity of spike pattern model increases progressively in Setups 2, 3, and 4. Furthermore, it is known that the performance of STDP-based rules degrades with a reduction in number of active afferents (Gütig and Sompolinsky, 2006; Masquelier et al., 2008). Thus, the success rate in these challenging setups can be significantly improved by increasing $N_{\mathrm{aff}\_\mathrm{active}}$, as demonstrated in Gautam and Kohno (2021) where $N_{\mathrm{aff}\_\mathrm{active}}$ was four to eight time larger.

**Table 4:** Comparison of the ideal model simulation with on-chip performance.

Setups	$N_{aff\_active}$/$N_{aff}$	Additional noise and jitter	Success rate (25% pattern appearance rate)		Success rate (10% pattern appearance rate)	
			Ideal Model	on-chip	Ideal model	on-chip
1	256/256	No	90%	96%	82%	90%
2	128/256	No	46%	80%	40%	(64%)*
3	256/256	Yes	64%	88%	60%	80%
4	128/256	Yes	8%	(26%)*	6%	(14%)*

### 3.4. Circuit parameters

In the experiments, only three parameters were modified across the setups: the resting membrane potential of the soma circuit, the initial value of synaptic efficacy, and the final value of $V_{\mathrm{LTD}}$ after adaptation. The actual values are listed in Table 5. The parameter $I_{\mathrm{rest}\_\mathrm{Vin}}$ is connected to one of the inputs of the differential pair of a wide-range transconductance amplifier circuit (Mead, 1989), whose output is connected to the membrane potential of the soma circuit. The other terminal of the differential pair is fixed at 500 mV. Parameter $I_{\mathrm{rest}\_\mathrm{Vin}}$ controls the current sourced from the transconductance circuit and is used to set the resting membrane potential of the soma circuit. The spiking threshold of the soma circuit is approximately 710 mV. Depending on the number of afferents encoding the spike patterns ($N_{\mathrm{aff}\_\mathrm{active}}$), the resting potential of the soma circuit varies across setups. The resting potential in Setups 1 and 3 (~ 870 mV) was higher than that in Setups 2 and 4 (~ 840 mV) because the number of active afferents ($N_{\mathrm{aff}\_\mathrm{active}}$) in the former setups was double that of the latter. Higher $N_{\mathrm{aff}\_\mathrm{active}}$ leads to the potentiation of a higher number of synapses. To compensate for this reduction in the number of available synapses in Setups 2 and 4, the resting membrane potential was reduced. Owing to the change in the resting membrane potential, the initial synaptic efficacy is also changed across setups. The efficacy value that caused the soma to spike in the desired frequency range of 40–200 Hz during the initial phase of the run was chosen Gautam and Kohno (2021). This criterion sets the range of synaptic efficacy values that can be selected. The third parameter, $V_{\mathrm{LTD}\_\mathrm{final}}$, which corresponds to the final value of $V_{\mathrm{LTD}}$ after adaptation, was adapted to different values to account for variations in the complexity of the setups. A high value of $V_{\mathrm{LTD}}$ implies a higher $t_{\mathrm{post}}$ (Figure 5B) and a higher probability of depression of synaptic efficacies. In Setups 1 and 2, $V_{\mathrm{LTD}\_\mathrm{final}}$ value of 829 mV was used. The inputs in Setups 3 and 4 contained additional noise and jitter (making pattern detection more challenging). Therefore, $V_{\mathrm{LTD}\_\mathrm{final}}$ was reduced to 817 and 813 mV, respectively. For the learning circuits, $V_{\mathrm{LTP}}$ was fixed at 780 mV and the initial value of $V_{\mathrm{LTD}}$ was 783 mV, which was then adapted to higher values during learning, as shown in Figure 5B.

**Table 5:** Parameters changed across setups.

Setups	$I_{rest\_Vin}$	Initial synaptic efficacy	$V_{LTD\_final}$
1	600 mV	5	829 mV
2	500 mV	4	829 mV
3	600 mV	6	817 mV
4	500 mV	4	811 mV

The rate at which $V_{\mathrm{LTD}}$ is adapted plays an important role in the learning process. In all runs, $V_{\mathrm{LTD}}$ reached its maximum value in approximately 100 s during the learning process. As the complexity of the input spike trains increases, a slowly rising $V_{\mathrm{LTD}}$ led to more stable learning. For example, a quicker adaptation rate where $V_{\mathrm{LTD}}$ reaches its maximum value in 40 s yields similar results in Setup 1, but the performance degrades in other setups, particularly in Setups 2 and 4 (with 10% pattern appearance rate). Delaying the maximum reaching time to more than 100 s did not result in any further improvement in performance. The shape of the adaptation curve is not important as long as it adapts slowly. Experiments were performed with linear, exponential, and stepwise increase in the value of $V_{\mathrm{LTD}}$ and similar results were observed.

### 3.5. Spiking latency

In the experiments, the parameters $V_{\mathrm{LTP}}$ and $V_{\mathrm{LTD}}$ were carefully selected. Compared to simulation-based studies (Masquelier et al., 2008; Gautam and Kohno, 2021), a smaller number of afferents were used in this study. This reduction affects the time at which the soma circuit spikes within the 50 ms long pattern after learning is completed. It is generally known that in the spike pattern detection tasks discussed in this study, the neuron learns to spike at the beginning of the 50 ms-long spike pattern (Song et al., 2000; Guyonneau et al., 2005). During the learning process, the neuron first spikes at a random point within the 50 ms-long spike pattern. During learning, the STDP-based learning rules potentiate those synapses that receive input spikes immediately before a postsynaptic spike. Stronger synaptic inputs advance the time of the postsynaptic spike in the next pattern presentation. This reduction in latency to spike within the pattern continues until the neuron learns to spike near the beginning of the pattern. When using the adaptive STDP learning rule, $V_{\mathrm{LTP}}$ (which sets $t_{\mathrm{pre}})$ and the number of afferents ($N_{\mathrm{aff}}$) influence the time at which the soma spikes within the 50 ms-long pattern. For any given value of$\;V_{\mathrm{LTP}}$, when the number of afferents is high, there is a higher probability of STDP learning to track back through the pattern by progressively potentiating synapses that were activated earlier in the pattern. However, this backtracking does not occur when the number of afferents is low (as is the case in this study). In the case of fewer afferents, backtracking can be achieved if the value of$\;V_{\mathrm{LTP}}$ ($t_{\mathrm{pre}}$) is increased. With a longer $t_{\mathrm{pre}}$,the learning rule allows the potentiation of temporally distant synapses in terms of their activation times. However, this also increases the probability of potentiating synapses not associated with the pattern, thereby making the learning process less stable and degrading overall performance. When $V_{\mathrm{LTP}}$ and the initial value of $V_{\mathrm{LTD}}$ were increased to 800 and 803 mV (from 780 and 783 mV, respectively, used to obtain the results in Table 2), the neuron learned to spike within 10 ms from the beginning of the pattern in all successful runs. However, the overall success rate decreased, particularly in the setups with 10% pattern appearance rate. Hence, relatively smaller values of the learning parameters $t_{\mathrm{pre}}$ and $t_{\mathrm{post}}$ (set *via* $V_{\mathrm{LTP}}$ and$\;V_{\mathrm{LTD}}$) were chosen to keep the learning process stable. When changing $V_{\mathrm{LTP}}$, the initial value of $V_{\mathrm{LTD}}$ must also be changed, because, according to the learning rule, the initial value of $t_{\mathrm{post}}$ (set by$\;V_{\mathrm{LTD}}$) must be close to $t_{\mathrm{pre}}$ (set by$\;V_{\mathrm{LTP}}$). It is noteworthy that even in Gautam and Kohno (2021), backtracking and spiking latencies under 10 ms were achieved in Setups 1 and 2, in which the number of afferents was high, but not in Setup 3, in which the number of afferents was low.

### 3.6. Power consumption

The average power consumptions (measured from the chip) of the soma circuit, 256 synapse circuits, and 256 learning circuits during the initial 50 s of the run (when the majority of synapse circuits were active and not depressed) were within 6 nW, 2.4 nW, and 2.1 μW, respectively. The power consumption measured from the learning circuit includes the consumption of up-down counters that store synaptic efficacies as well as additional circuitry measuring the spike timings. The static power consumption (when the afferents were inactive) of the 256 synaptic circuits and 256 learning circuits was within 120 pW and 200 nW, respectively.

## 4. Discussion and conclusion

This study focused on the neuromorphic implementation of the adaptive STDP rule with 4-bit synapses. The circuit implementation is simpler than that of conventional STDP circuits with the same resolution. Both rules require a circuit to measure the time between the pre-and postsynaptic spikes. However, the circuit used to update the efficacy is much simpler in the adaptive STDP rule, primarily because a rectangular learning window was used instead of an exponential one, and the efficacy update at any time instant was restricted to a single bit. Thus, the update can be performed using a simple 4-bit up-down counter circuit, thereby eliminating the need for additional circuits, such as adders, subtractors, and analog-to-digital converters (ADCs), which are required to implement the STDP rule. The overhead of the additional circuit required to implement the adaptation of $V_{\mathrm{LTD}}$ ($t_{\mathrm{post}}$) is negligible because it can be shared by all the synapses. Without the adaptation of $V_{\mathrm{LTD}}$, even if a higher synaptic resolution (8-bit) is used with the rectangular learning window, spike pattern detection is not successful. In Cassidy et al. (2011), such a learning function ($t_{\mathrm{post}}$>$t_{\mathrm{pre}}$) with 8-bit synapses was shown to achieve a bimodal distribution of efficacies in a balanced excitation experiment (Song et al., 2000). With 8-bit synapses and spike pattern model used in this study, we also observed a bimodal distribution of synaptic efficacies after learning. However, pattern detection was not successful because of the presence of many false alarms even after 450 s of learning.

The performance was evaluated and compared using a biologically possible input spike train model with embedded spike patterns. This input model was chosen because its spike trains and embedded patterns are built on biologically plausible prerequisites, making them suitable for networks that explore biologically plausible computations.

The adaptive STDP rule was proposed in Gautam and Kohno (2021), where numerical simulation results with ideal models were shown. However, in such simulations, the various effects in analog VLSI, such as device mismatch, parasitics, and thermal noise, cannot be fully considered. The experimental results demonstrate that, even in the presence of these effects, the performance of adaptive STDP learning is either similar to or better than that obtained in the numerical simulation (Table 4). Additionally, we also observed unstable results in (more challenging) Setups 2 and 4, where, owing to thermal noise, for the same input spike trains, certain runs succeeded in detecting the patterns whilst others failed. Such unstable spiking is known to occur in the brain but cannot be observed in ideal numerical simulations unless additional noise is added. Surprisingly, the performance on-chip was better than the ideal model simulation in all setups. One probable reason for this can be attributed to thermal noise as it might contribute to suppression of false alarms by disturbing the postsynaptic spike’s timing. Such suppression is not possible in ideal model simulation unless additional noise is incorporated. This hypothesis will be validated in future works. The integrated circuit fabricated in this study was scalable. Learning occurs in an on-chip and completely unsupervised manner. To the best of our knowledge, this is the first neuromorphic chip to accomplish spatiotemporal spike pattern detection in noisy inputs using a low-bit-resolution synaptic memory in an unsupervised regime.

The resolution of the synaptic efficacy in our synaptic circuit is 4-bit. A 4-bit DAC was used to generate a synaptic current corresponding to the synaptic efficacy. In contemporary synapse circuits, these DACs are designed using large transistors or current-mirror circuits to minimise the device mismatch, which increases the silicon area and power consumption (Wang and Liu, 2006; Moradi and Indiveri, 2014). Instead of focusing on the accuracy of the DAC, the DAC in our synaptic circuit was designed using relatively smaller transistors (see the caption of Figure 4) without any mirroring circuits, which saves significant silicon area and power. The DAC has monotonicity (differential nonlinearity (DNL) > −1); however, linearity is not guaranteed. Device mismatch also affects the amplitude and time constant of synaptic currents, which are controlled by the voltage parameters$\;sV_{w}$ and$\;sV_{t}$, respectively. The learning parameters $t_{\mathrm{pre}}$ and $t_{\mathrm{post}}$ are controlled by the voltage parameters $V_{\mathrm{LTP}}$ and$\;V_{\mathrm{LTD}}$, respectively. These voltages are common to all synapses and learning circuits. The fact that adaptive STDP learning worked well with such a DAC demonstrates that a low-resolution (4-bit) and relatively low-accuracy DAC is sufficient for its implementation. The effects of DAC accuracy and device mismatch on the pattern-detection performance should be evaluated in the future.

**Figure 9:**
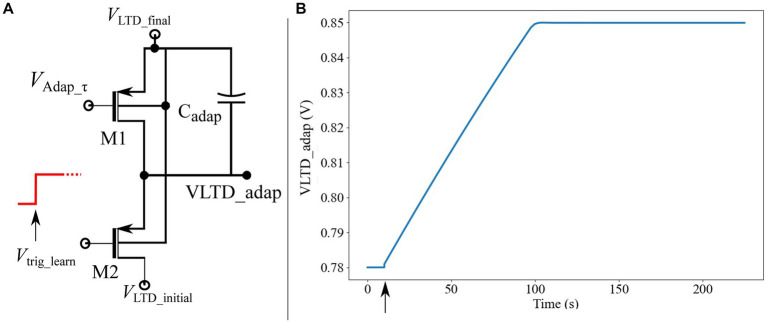
**(A)** Circuit to generate a smoothly rising adaptation curve. **(B)** Adaptation curve plotted using Spectre simulation of circuit in Figure 9**A**. The learning begins when the afferent synapse circuits are activated (marked by an arrow).

In this study, the adaptation curve was generated using an external voltage source. In future, it will be integrated into the chip. A low-power circuit that generates a smoothly increasing adaptation curve is shown in Figure 9A. Spectre Simulator was used to plot Figure 9B with voltages $V_{\mathrm{LTD}\_\mathrm{inital}}$ and $V_{\mathrm{LTD}\_\mathrm{final}}$ of 780 and 850 mV, respectively.

Another important parameter is$\;V_{\mathrm{LTD}\_\mathrm{final}}$. An excessively high value causes the neuron to stop spiking (owing to the depression of the majority of synapses), and an exceedingly small value results in many false alarms. False alarms are not necessarily harmful in multi-layer networks. When occurring stochastically, they can contribute as the background population activity to maintain the membrane potential of the neurons in the next layer close to their spiking threshold. Thus, in addition to the features learned in the first learning layer (spikes occurring within the pattern), the parameter $V_{\mathrm{LTD}\_\mathrm{final}}$ can be used to control the rate of stochastic spikes serving as inputs to successive layers. This aspect should be explored in future studies.

A bidirectional current conveyor circuit was used as an interface to transmit the synaptic current into the soma circuit. The current conveyor circuit induced noise in the soma circuit. The power supply lines had extremely low ripple noise because ultralow ripple power supplies were used. A plausible reason for this noise is the thermal noise in the silicon and bias voltage sources. The induced noise caused the membrane potential of the soma circuit to fluctuate randomly by approximately 50 mV (peak-to-peak) at a resting membrane potential of 800 mV. In addition, the amplitudes of the fluctuations increased as the resting membrane potential approached the spiking threshold (710 mV). To minimise this noise, voltages $V_{\mathrm{dd}\_\mathrm{out}}$ and $V_{\mathrm{ss}\_\mathrm{out}}$, which are the power and ground terminals of the output branches of the current conveyor, respectively, were fixed at 954 mV and 50 mV (instead of their original values in the Spectre simulation of 1 V and 0 V). With this change, the random fluctuation in membrane potential was reduced to approximately 30 mV at a resting membrane potential of 800 mV. To reduce noise further, the resting membrane potential of the soma circuit was maintained at 850 mV (approximately 150 mV from the spiking threshold). The static power consumption of the current conveyor circuit was under 90 nW (evaluated using Spectre simulation). In the future, the current conveyor design will be improved to minimise the induced noise so that the resting membrane potential of the soma circuit can be maintained close to the spiking threshold. An improved circuit may also improve the on-chip performance of the spike pattern models in Setups 2 and 4.

Several mixed-signal neuromorphic chips with STDP learning capabilities have been proposed to date. Although analog STDP circuits that store synaptic efficacy on capacitors offer higher energy efficiency compared to the mixed-signal circuits (like the one used in this study), they are often impractical in many applications due to the need for large capacitors and their susceptibility to leakage issues. For an extensive review of such circuits, please refer to (Azghadi et al., 2014b). The BrainscaleS (Schemmel et al., 2010) and BrainscaleS2 (Pehle et al., 2022) chips use 4- and 6-bit synaptic efficacies and were implemented in 180 nm and 65 nm technology nodes, respectively. Its circuits for tracking the pre-and postsynaptic spike traces are purely analog, which store the state of these traces as voltages on a capacitor. A high-speed ADC serially reads these voltages and transfers them to a digital Plasticity Processing Unit (BrainscleS2) that updates the synaptic efficacy. The plasticity module in ROLLS (Qiao et al., 2015) and, more recently, Dynap-SEL (Moradi et al., 2018) chips from INI implement the spike-dependent synaptic plasticity (SDSP) learning rule and use similar learning circuits (Brader et al., 2007). The synaptic resolution in the Dynap-SEL chip is increased to 4 bits (Thakur et al., 2018), whereas the ROLLS chip uses a ~ 1.5-bit palimpsest synapse. The ROLLS chip is implemented in a 180 nm node and Dynap-SEL in both the 180 nm (Moradi et al., 2018) and 28 nm fully depleted silicon on insulator (FD-SOI) technology nodes (Qiao and Indiveri, 2016). Our current chip is fabricated in a 250 nm technology node and comprises a single neuron circuit. Thus, its performance cannot be appropriately compared with those of these chips. However, certain differences can be highlighted. The circuits in the BrainscaleS project were designed to operate in the above-threshold domain (of MOS transistors) with accelerated timescales, whereas our circuit was designed to operate in biological timescales. Thus, for the same technology node, the area occupied by the learning circuits to calculate the traces of pre-and-post synaptic spikes in our chip would be higher than that in the BrainscaleS(2) chip, but the power consumption would be significantly lower. We could not find the exact value of the power consumption of the learning circuit. The SDSP rule implemented in the ROLLS and Dynap-SEL chips is a rate-based semi-supervised learning rule that classifies input spikes based on their spike rates (Boi et al., 2016; Kreiser et al., 2017). Amongst the spike-based learning circuits proposed thus far, the bistable palimpsest synapse in the ROLLS chip is the most efficient in terms of area and power. In our chip, the energy consumed to process a pair of pre -and postsynaptic spikes and update the synaptic efficacy by 1-bit is about 235 pJ. This value was significantly higher than that of the ROLLS chip (77fJ). However, its performance is limited by its low-resolution efficacy (Pfeil et al., 2012; Saxena et al., 2017). Its limitations are discussed in Boi et al. (2016). The newer Dynap-SEL chip will have better performance because it uses 4-bit synaptic efficacy; however, its power consumption will also be significantly higher because it also uses a counter circuit similar to our implementation to update synaptic efficacy. We could not find the exact value of the power consumption for the learning circuit in the Dynap-SEL chip. In contrast to the rate-based SDSP rule implemented in the ROLLS and Dynap-SEL chips, the learning mechanism in our chip is driven by the spike timings of pre- and postsynaptic neurons. The input spike trains have a uniform average rate inside and outside the spike patterns, and only the spike timing relationship amongst the afferents differentiates the spike pattern from the noise. Thus, in this study, the information was not coded according to spike rate. In addition, the learning mechanism is devoid of a teacher signal; that is, it is completely unsupervised, in which a spatiotemporal spiking pattern buried in noise can be spontaneously detected. Since a major part of our learning circuit is digital, it can be easily scaled down to minimise the silicon area when implemented in lower technology nodes.

The power consumed by the fabricated learning circuitry in the experiments was higher than that evaluated using the Spectre simulator. It can be attributed to variations in the fabrication process (Mead, 1989). The static power consumption of 256 learning circuits was less than 200 nW, which scales to less than 800 pW for a single learning circuit. In Spectre simulator, this value for a single learning circuit was under 140 pW in the typical process corner but under 1.4 nW in the worst power corner, thereby implying that the chip was fabricated away from the typical process corner. The major contributors to dynamic power consumption in the learning circuit is the short-circuit currents during the switching of the inverters. Proven techniques, such as the use of starved inverters, will be incorporated in the future to minimise the dynamic power consumption. Power consumption can be further minimised by implementing circuits in FD-SOI technology, which has comparatively lower leakage currents.

Most mixed-signal neuromorphic chips employ the nearest-neighbour pair-based STDP rule. However, different variants of the STDP rules that incorporate multi-spike interactions have been observed in different regions of the brain (Wang et al., 2005; Froemke et al., 2006; Pfister and Gerstner, 2006; Bono and Clopath, 2017). Within the analysis by the synthesis neuroscientific framework, a significant challenge for neuromorphic researchers is to develop scalable and hardware-friendly learning circuits that consider such multi-spike interactions and use SRAM or novel non-volatile memory devices to store synaptic efficacies. The design of these learning circuits will be explored in future studies.

The chip architecture is scalable for the incorporation of multiple neuron circuits. Our future chips will be designed in a lower-technology node and integrate multiple neuron circuits. Event-based communication circuits will be expanded in line with the Address Event Representation (AER) protocol that has been successfully implemented in many large-scale neuromorphic chips (Thakur et al., 2018). The chip in this study was fabricated in a relatively older TSMC 250 nm technology node, but all the circuits presented in the study were fitted to lower technology nodes; 250 nm was chosen because of its availability and financial constraints. We plan to use the 28 nm FD-SOI technology in future work.

## Data availability statement

The original contributions presented in the study are included in the article/supplementary material, further inquiries can be directed to the corresponding author.

## Author contributions

AG performed the study. TK supervised it. All authors contributed to the article and approved the submitted version.

## Funding

This study was partially supported by JSPS KAKENHI Grant Number 21H04887, DLab, The University of Tokyo in collaboration with Cadence Design Systems, Inc., and JST SICORP Grant Number JPMJSC15H1.

## Conflict of interest

The authors declare that the research was conducted in the absence of any commercial or financial relationships that could be construed as a potential conflict of interest.
